# Morphological Divergence of Hermann’s Tortoise (*Testudo hermanni boettgeri* Mojsisovits, 1889) in Albania

**DOI:** 10.3390/ani11010134

**Published:** 2021-01-09

**Authors:** Sokol Duro, Bektaş Sönmez, Ozan Gündemir, Tefik Jashari, Tomasz Szara

**Affiliations:** 1 Faculty of Veterinary Medicine, Agricultural University of Tirana, 1029 Tirana, Albania; durosokol@ubt.edu.al; 2 Suşehri Timur Karabal Vocational School, Sivas Cumhuriyet University, 58600 Sivas, Turkey; bsonmez@cumhuriyet.edu.tr; 3 Department of Anatomy, Faculty of Veterinary Medicine, Istanbul University-Cerrahpasa, 34500 Istanbul, Turkey; ozan.gundemir@istanbul.edu.tr; 4 Institute of Graduate Studies, Istanbul University-Cerrahpasa, 34500 Istanbul, Turkey; tefikjashari13@gmail.com; 5 Department of Morphological Sciences, Institute of Veterinary Medicine, Warsaw University of Life Sciences-SGGW, 02-776 Warsaw, Poland

**Keywords:** Bergmann’s rule, carapace morphometry, plastron scutes, Hermann’s tortoises, *Testudo hermanni boettgeri*

## Abstract

**Simple Summary:**

The morphology of chelonians provides basic information about development, evolution, biodiversity, biomechanics, behavior, ecology, and physiology. Furthermore, it has also played an important role in characterizing populations and analyzing the similarities between populations. This study investigates the morphological variation of Hermann’s tortoise (*Testudo hermanni boettgeri* Mojsisovits, 1889) between specimens from five different populations in Albania. It also provides basic data on the morphological characteristics of the Hermann’s tortoise. Hermann’s tortoise in the Albanian population were regionally diverged into three different populations that were situated in northern (Shkodra), central (Tirana, Berati, and Ballshi), and southern (Saranda) Albania. Moreover, female individuals were larger and heavier than male individuals, and the females followed Bergmann’s rule. The morphological divergences may be due to molecular variations or environmental conditions of the regions.

**Abstract:**

Testudines show phenotypic plasticity, and variation among specific populations within a species is widespread. Morphological differences between populations may reflect ecological factors that drive adaptation to local conditions. In this context, we gathered basic data on the morphology of the Hermann’s tortoise (*Testudo hermanni boettgeri* Mojsisovits, 1889) to document their variation across different geographical regions. We surveyed Hermann’s tortoises in five different locales within Albania during April and May 2020 and measured 20 morphological characteristics, including carapace and plastron dimensions. We measured 188 tortoises (81 males, 107 females) in this study, and females were larger (*p* = 0.0001) and heavier (*p* = 0.0001) than males. Mean straight carapace length (SCL) and body mass were 172.4 mm and 1128.8 g, respectively, for females, and 151.3 mm and 735 g, respectively, for males. The Albanian *T. h. boettgeri* were regionally diverged into three different populations that were situated in northern (Shkodra), central (Tirana, Berati, and Ballshi), and southern (Saranda) Albania. The body size (curved carapace length (CCL)) of females was positively correlated (*r* = 0.216; *p* = 0.025) with the latitude, in accordance with Bergmann’s rule. However, there was no correlation between body size and latitude in males. These striking regional differences among Albanian *T. h. boettgeri* strongly suggest that further study of molecular variations and reproductive output of Hermann’s tortoises is warranted.

## 1. Introduction

Hermann’s tortoise (*Testudo hermanni*) is a terrestrial species that is widespread in the European Mediterranean region. It is one of the herpetofauna species of Albania, which can be found in an area ranging from the shores of the sea to an altitude of approximately 1300 m in the mountains. However, most populations of Hermann’s tortoise are found below 500 m [1,2,3]. The most common habitats of the Hermann’s tortoise are agricultural lands, canals, hilly grasslands, areas of sparse vegetation, and land near forests. The species is listed globally as ‘‘near threatened’’, according to the International Union for Conservation of Nature [4]. Due to the overuse of tortoises for the pet trade, serious declines in their populations have been observed [5]. In fact, the Hermann’s tortoise constitutes 13% of the world *Testudo* trade [6], and is listed by the Bern Convention and European Union’s Habitat Directive as a species in need of strict protection. International trade of the species is regulated by the Convention on International Trade in Endangered Species of Wild Fauna and Flora [7]. The existence of *T. hermanni* is threatened by many additional factors such as rapid urbanization and concomitant habitat loss, climate change, increasing ambient temperatures, multiple summer fires, prolonged droughts or floods, and increased human activity [8].

Hermann’s tortoises are only found in Europe, the Balkans, and the Turkish Thrace. Based only on external morphology, there are two recognized subspecies: *Testudo hermanni hermanni* and *Testudo hermanni boettgeri* [9,10]. While *T. h. hermanni* inhabits the western part of the Po Valley in Italy, *T. h. boettgeri* inhabits the Balkans and Turkish Thrace [7]. The tortoise found in Albania belongs to the subspecies Eastern Hermann’s tortoise (*T. h. boettgeri*), which possesses external morphological features characteristic for the species, such as light yellow external coloration of the carapace; black pigmentation of the plastron that is less defined than the bands (i.e., discontinuous) and in some cases discolored; length of the interpectoral suture that is greater or equal than the interfemoral suture; the presence of inguinal scutes on either side of the shell in almost 100% of all individuals [11].

The carapace is high and almost oval in form in females and sub-trapezoidal in males. In the middle line of the carapace, there is a nuchal (cervical) scute, five vertebral scutes, four pleural scutes more laterally on either side, and twelve marginal scutes surrounding the first. In the plastron, the scutes are nominated from the cranial part as gular, humeral, pectoral, abdominal, femoral, anal, and inguinal scutes [12,13,14].

The morphology of the chelonian shell can provide some detailed information about reproduction, locomotion, and protection against predators [15]. The overall shell shape in turtles and tortoises affects fecundity selection in females and sexual selection in males [16,17]. In fact, their shell morphology consists of a continuous set of sutured shell plates and free body parts covered with horny scales that can be measured individually and precisely, which play a role in mating and non-mating activities. The adult body sizes in turtles offer a means to estimate the relationship between growth rates and body shape [18]. Furthermore, total body size is also an important morphological feature affected by ecological and sexual selection in chelonians [19,20] and can show geographic diversity [16,21,22].

Phenotypic plasticity and variation in specific populations is a widespread phenomenon in *Testudo* species [23,24]. For instance, differences in body size of *T. h. boettgeri* populations in Serbia and Montenegro have been reported [25]. Similarly, *T. h. boettgeri* populations in Trebinje (a municipality in Bosnia and Herzegovina) and the Pčinja river valley (Serbia) in the Balkan Peninsula showed variation in carapace shape [26]. Hermann’s tortoise populations in the Republic of Serbia and the former Yugoslav Republic of Macedonia showed morphological differences [15]. The size and shape of Italian Hermann’s tortoises varied according to a north-south cline, following Bergmann’s rule that proposed a positive relationship between mean body size and latitude [22]. Similarly, the carapace length of *Testudo graeca* was correlated with latitudes globally and locally [27], also following Bergmann’s rule. Moreover, morphological divergence was also found in *T. graeca* populations from west-central Morocco [28]. In contrast, the populations of *T. graeca* along the Mediterranean coast of Turkey were morphometrically homogenous [29]. Adult eastern populations of Hermann’s tortoises of the Balkans exhibited long carapace length (>180 mm in females and >155 mm in males) [10]. Specimens with the longest carapace length were found in Bulgaria [30], whereas those with the shortest length were found in the south of the Peloponnese in Greece [31].

Morphological differences among populations give scientists clues about ecological and biological factors that drive adaptation. They can also help researchers gain insights into locomotion, fecundity and sexual selection, and the health conditions of populations. Although studies on the morphological differences among Hermann’s tortoise populations have been conducted in several countries in the Balkan peninsula, only one study of *T. h. boettgeri* has been conducted for the Albanian population in southern Albania (Vlora), with a small sample [8]. Therefore, the aim of our investigation was to collect and analyze basic data on the morphological characteristics of the Hermann’s tortoise in Albania and to assess any potential morphological variation between specimens from five different regions.

## 2. Materials and Methods 

### 2.1. Study Area

**Figure 1:**
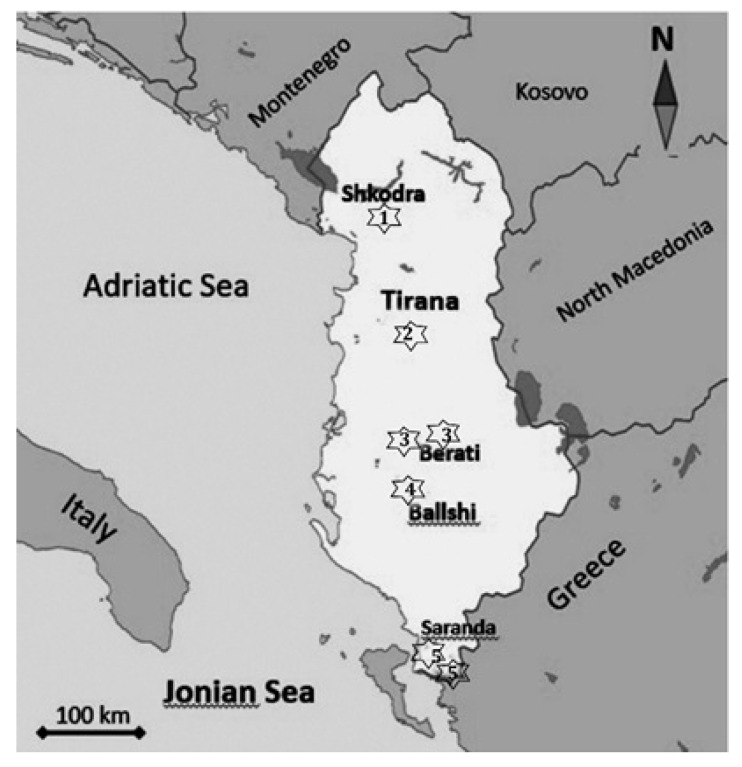
Location and distribution of the field survey regions of Hermann’s tortoises in five regions of Albania.

The study was conducted in the hills and mountains of five Albanian regions, distributed from north to south and from the sea to the middle of the country, during spring (April and May) of 2020. These regions were designated using the name of the major city located within or near the study area, such as Shkodra, Tirana, Berati, Ballshi, and Saranda (Figure 1).

The Shkodra region lies 110 m above sea level and has a Mediterranean climate with hot summers with continental influences. The average yearly temperature varies from 14.5 to 16.8 °C. The average yearly precipitation is about 1700 mm (from 34.1 mm in July to 229.8 mm in November [32]), which makes the area one of the wettest in Europe. The samples were collected from the rural areas of Maraç (41.9765831° N, 19.6661893° E). 

The Tirana region has an average altitude of 521 m above sea level, with a humid subtropical climate. Temperatures vary throughout the year from an average of 6.7 °C in January to 24 °C in July. The average precipitation is about 1266 mm per year, and the humidity ranges from 62 to 74% [32]. The samples were collected from the rural areas of Baldushk (41.1957547° N, 19.8395281° E). 

The Berati region lies an average of 53 m above sea level but contains hills from 130 to 200 m above sea level. Its climate is classified as warm and temperate. The average temperature is 15.7 °C, ranging from 7.4 °C (in January) to 24.4 °C (in July and August). The average precipitation is 1032 mm annually [32]. The samples were collected from the rural areas of Ura Vajgurore (40.7652882° N, 19.8847051° E) and Lumas (40.812114° N, 20.0032559° E).

The Ballshi region lies 500 m above sea level. The average temperature for the last 30 years has been 15.8 °C, ranging from 5.8 °C in January to 24.5 °C in July. The average annual precipitation is 1041 mm [33]. The samples were collected from the rural areas of Aranitas (40.5977479° N, 19.8089151° E) in the Ballshi region. 

The Saranda region lies almost entirely at sea level but contains some land as high as 300–500 m above sea level. Saranda has a typical Mediterranean climate with dry and hot summers and mild and wet winters. The average temperature is 15.6 °C, which ranges from 1 °C during winter to 28 °C during summer. The average precipitation is 1370 mm annually [32]. The samples were collected from the rural areas of Çukë (39.8276987° N, 20.0610213° E) and Konispol (39.6592742° N, 20.1804537° E).

### 2.2. Data Collection and Measurements

Ethical approval for this study was obtained from the Ethics Commission of the Veterinary Faculty of Tirana, Albania (No. 179/20.05.2020). We randomly located and collected by hand a total of 188 healthy and undamaged tortoises and evaluated them in their native wild habitat. All Hermann’s tortoises were evaluated carefully for external morphologic characteristics to determine the correct sex and subspecies. Sex was determined by the concavity of the plastron, tail length, and curve of supracaudal scutes. These characteristics were found to be more prominent in male tortoises than in female tortoises [13,14,25]. After measurements were recorded, tortoises were released at the same locations where they were captured. We used an electronic scale (1 g error rate) to measure body mass, and a digital caliper (precision 0.01 mm) and tape measure (1 mm error) for external body measurements.

**Figure 2:**
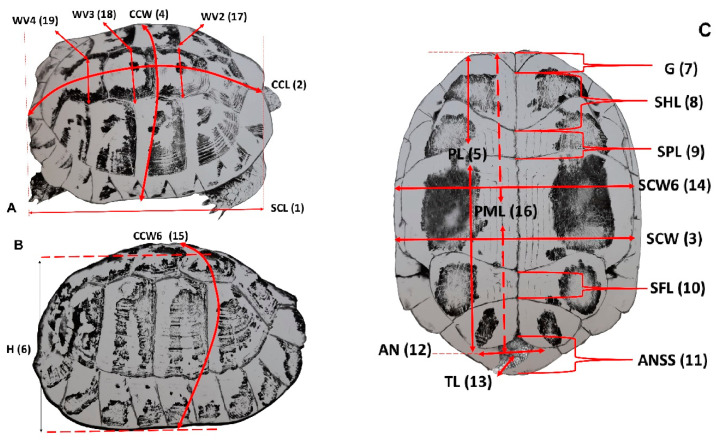
Measurement parameters of the carapace and plastron of *T. h. boettgeri*. Abbreviations are defined in the Materials and Methods section. The numbers in parentheses indicate the parameter number. (**A**,**B**) indicate carapace measurements, (**C**) indicates plastron measurements.

We evaluated 19 external measurements (shown in Figure 2) as previously reported [6,28]. The morphometric dimensions recorded were 1: straight carapace length (SCL), 2: curved carapace length (CCL), 3: straight carapace width (SCW), 4: curved carapace width or maximal perimeter side to side (CCW), 5: plastron length (PL), 6: maximal high (H), 7: intergular suture length (G), 8: interhumeral suture length (SHL), 9: interpectoral suture length (SPL), 10: interfemoral suture length (SFL), 11: distance from anal notch to supracaudal scute tip (ANSS), 12: anal notch width between two anal scutes (AN), 13: tail length from cranial margin of cloaca to the tail tip (TL), 14: carapace width at the level of the sixth marginal scute (SCW6), 15: curved carapace width at the level of the sixth marginal scute (CCW6), 16: plastron midline length from gular notch to anal notch (PML), 17: maximal width of second vertebral (WV2), 18: maximal width of third vertebral (WV3), and 19: maximal width of fourth vertebral (WV4) as well as body mass.

Body condition index (CI) in the tortoise was calculated according to the standard procedures given in similar studies [34,35] as the ratio of observed body mass (M) to the predicted body mass (M’) obtained from the linear regression of body mass to SCL. CI was expressed as log (M/M’), where M was observed mass and M’ was mass estimated from SCL. A previous study [34] found that the log (M/M’) represented the best body condition index (CI) based on equal to residuals from the regression of log M on log L [34]. This ensured that the distribution of log (M/M’) did not deviate from normality and allowed the analysis of interaction effects in the analysis of variance. This situation was checked with the Shapiro–Wilk normality test for both sex and regions (*p* > 0.05).

### 2.3. Statistical Analysis

Differences in body mass (M) and SCL between female and male tortoises were investigated by independent sample *t*-test. Differences in CI between regions were performed by a univariate test with the general linear model. Moreover, differences between male and female CI were analyzed with one-way ANOVA [7,35].

Multivariate comparisons of morphological dimensions between regions and sexes were performed with multivariate analysis of covariance (MANCOVA), using SCL as a covariate [28]. The equality of covariance matrices of dependent variables between groups was tested with Box’s M test, and deviations from normality and homogeneity of covariance matrices were found (*p* < 0.005). Therefore, Pillai’s trace test was used as the most robust standard test in multivariate comparisons for deviations from normality and equality of covariance matrices [36,37]. The relationship between mean body size and latitude for north-south cline was tested with Spearman’s rho (two-tailed test) [27].

The degree of similarity among regions and sex were assessed by discriminant functional analysis (DFA) with stepwise selection (F for entry: 3.84; F for removal: 2.71) [6,28]. Population centroids with 95% confidence ellipses derived from the DFA were used to visualize relationships among the individuals of regions. Correct classification of regions into their groups were tested with the cross-validation test in DFA (regions were assigned to the samples using the canonical functions). This output shows the number of cases correctly and incorrectly assigned to each region based on discriminant analysis. The percentage of correctly classified individuals gives a measure of the morphological distinctness of the samples. The number of misclassified individuals indicates the degree of intermingling between populations.

All statistical analyses were performed with IBM SPSS Statistics 20 software, and 95% confidence ellipses graphs were generated using XLSTAT (Addinsoft, version 2014.05.3). All means were presented with standard deviation (±) and minimum and maximum.

## 3. Results

A total of 188 adult Hermann’s tortoises were collected from five regions in Albania. The sex ratio of the *T. h. boettgeri* sample we studied was female-biased, with 1.3 females per male (107 females, 81 males). Distribution of tortoises by region was 28.2% in Berati, 26.1% in Saranda, 21.3% in Tirana, 13.3% in Ballshi, and 11.2% in Shkodra. Detailed descriptive statistics of morphological dimensions by sex and region are shown in Supplementary Table S1.

The mean body mass was 735 ± 241.8 g (range: 160–1250 g) for males and 1128.8 ± 355.6 g (range: 250–1920 g) for females, with the peak frequency distributions ranging between 800 and 1000 g (28.4%) for males and 1000 and 1200 g (32.7%) for females. The mean SCL was 151.3 ± 18.2 mm (range: 100–190 mm) for males and 172.4 ± 22.9 mm (range: 100–220 mm) for females, with the peak frequency distributions ranging between 139 and 152 mm (30.9%) for males and 178 and 191 mm (27.1%) for females. Females were heavier (t = −8.57, df = 186, *p* = 0.0001) and larger (t = −6.81, df = 186, *p* = 0.0001) than males. 

There was no correlation (*p* > 0.05) between mean body size (SCL and CCL) and latitude for north-south cline in males, rejecting Bergmann’s rule. On the other hand, the CCL of females was positively correlated with north latitude degrees (*n* = 107, *r* = 0.216, *p* = 0.025), following Bergmann’s rule.

**Figure 3:**
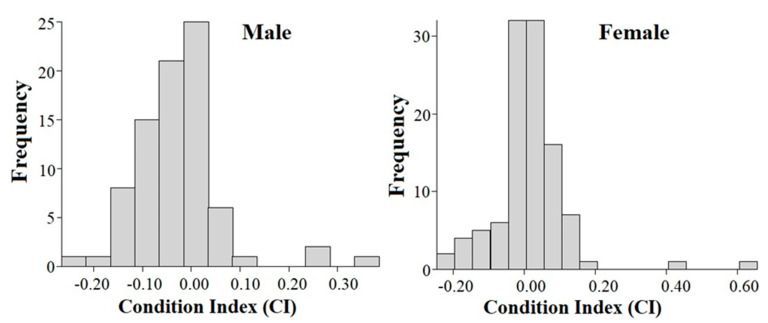
Frequency distributions of body condition index (CI) in male (*n* = 81) and female (*n* = 107) Hermann’s tortoises in Albania.

The body condition index (CI) of Hermann’s tortoises ranged from −0.24 to 0.62, with a mean CI of 0.00 in all regions. A mean CI of 0 indicates that the observed body mass is equal to the predicted body mass. Descriptive statistics of CI by region are shown in Table 1. The mean CI differed (F = 2.612, df = 4, *p* = 0.037) among regions. The CI was positive in the Tirana and Berati regions, whereas it was negative in the other regions. The mean CI (F = 10.492, df = 1, *p* = 0.001) was influenced by sex and ranged from −0.23 to 0.36 (mean of −0.02 ± 0.08) in males and from −0.22 to 0.61 (mean of 0.01 ± 0.10) in females. The frequency distribution of CI by sex is illustrated in Figure 3.

When differences among regions were tested using SCL as a covariate in MANCOVA, differences were observed (Pillai’s trace = 2.302, F = 10.274, *p* = 0.001). In addition, differences were also found for males (Pillai’s trace = 2.982, F = 8.013, *p* = 0.001) and females (Pillai’s trace = 2.242, F = 5.035, *p* = 0.001) among the regions. 

**Figure 4:**
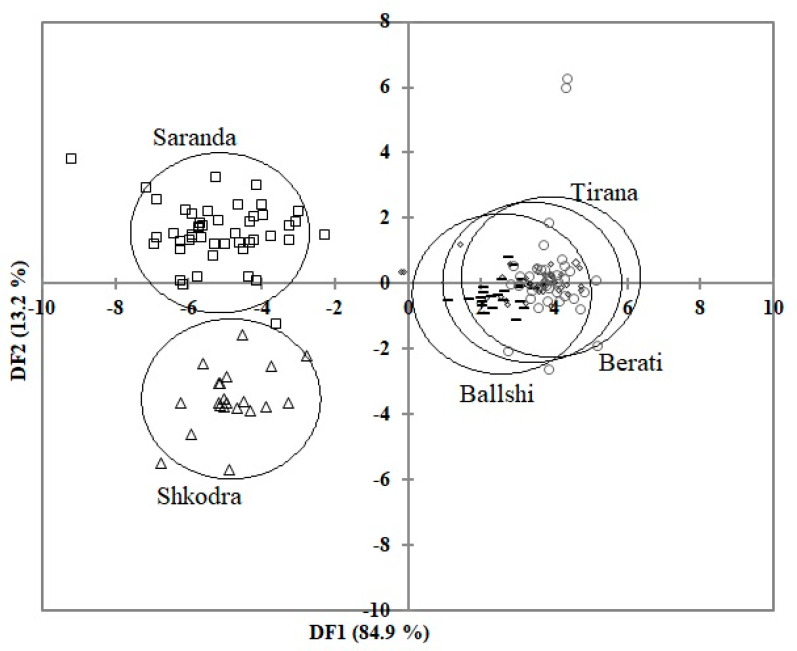
Discriminant function analysis plot with 95% confidence ellipses based on morphological analysis among the five regions in Albania.

In discriminant function analysis, the first canonical function accounted for the largest amount of between-group variability (84.9%), while the second, third, and fourth accounted for 13.2, 1.4, and 0.5%, respectively. DF1 and DF2 explained 98.2% of the between-group variation and revealed clear between-population differences. The Saranda and Shkodra populations were the most morphologically isolated from each other and from all other samples (Figure 4), suggesting that there is limited intermingling between these Saranda and Shkodra populations. With respect to correct initial classification of individuals into their original populations, 83.5% of original classifications were correct (Table 2). Not unexpectedly, the proportion of Saranda and Shkodra samples correctly classified into their original group was the highest (100%).

In order to discern which morphometric characters differentiate populations, pooled within-group correlations between discriminating variables and standardized canonical discriminant functions were produced with DFA. This analysis revealed that the observed differences were mainly carapace dimensions, such as SCW and CCW, followed by (in order of decreasing influence) body mass, PL, G, SFL, AN, TL, SCW6, and WV2.

**Figure 5:**
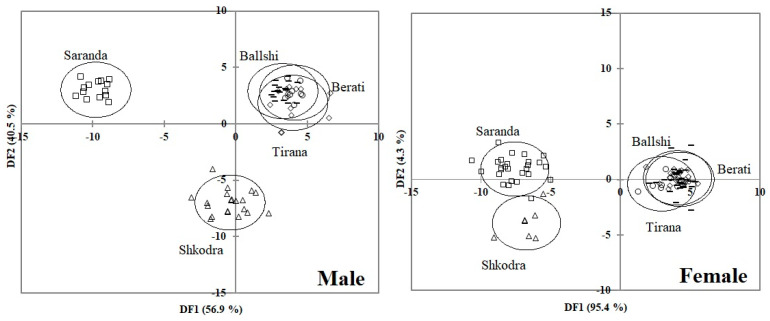
Discriminant function analysis plot with 95% confidence ellipses based on the morphological analysis by the male and female among the five regions in Albania.

Discriminant function analysis by sex among the regions was also performed. DF1 (56.9%) and DF2 (40.5%) explained 97.4% of the between-group variation and revealed clear population differences by region in males. The Saranda and Shkodra populations were the most isolated from each other and from all other samples (Figure 5). The initial classification of individuals into their proper population was correct in 90.1% of cases. The proportion of samples initially classified into their correct group was the highest (100%) in Saranda, Shkodra, and Ballshi, while Tirana was 78.6% and Berati was 61.5% correctly classified initially. The observed differences were mainly from carapace dimensions, such as CCL, SCW, and CCW, followed by PL, AN, TL, and SCW6.

Plotting DF1 (95.4%) and DF2 (4.3%) explained 99.7% of the between-group variation and revealed clear differences between populations by region in females. The Saranda and Shkodra populations were the most isolated from each other and from all other samples (Figure 5). Initially, 78.5% of samples were classified correctly into the proper group. The proportion of samples initially correctly classified was highest (100%) for the Saranda and Shkodra samples, followed by Tirana (92.3% correct initial classification), Ballshi (71.4%), and Berati (52.5%). The observed differences were mainly from carapace dimensions, such as SCW and CCW, followed by SFL and SCW6.

## 4. Discussion

This was the first study of Hermann’s tortoises with samples obtained from nearly all regions of Albania, and it was the largest-ever study of Hermann’s tortoises in Albania (188 specimens studied). The overall population studied was female-biased, similar to the only other study conducted in Albania [8]. A female-biased population was also reported in Serbia and the former Yugoslav Republic of Macedonia [15], as well as in the central Balkans [25]. In contrast, a male-biased population was observed in Greece [38] and in Montenegro of the former Yugoslavia [39]. These differences in sex bias among the various studies may be due to differences in maturity timing between the sexes [40], differences in mortality rates of the sexes [41], or some unquantified environmental influence, such as high ambient temperature or aberrant rainfall patterns.

The mean body mass of Albanian Hermann’s tortoises was 735 ± 241.8 g (range: 160–1250 g) for males and 1128.8 ± 355.6 g (range: 250–1920 g) for females, and the mean SCL was 151.3 ± 18.2 mm (range: 100–190 mm) for males and 172.4 ± 22.9 mm (range: 100–220 mm) for females. Females were significantly larger and heavier than males, and this was consistent with previous studies [7,15,25,42]. In the Vlora region of Albania, males Hermann’s tortoises were 130–180 mm, and females were 150–200 mm [8]. These measurements were similar to our results for Albania overall but smaller than tortoises found in the study of eastern populations of adult Hermann’s tortoises of the eastern Balkans that had an average carapace length of more than 180 mm in females and more than 155 mm in males [10]. The Hermann’s tortoises of Limnaji (Montenegro) were similar in size (151 mm for males and 165 mm for females) to the Albanian tortoises, but the population on the island of Starčevo (Montenegro) was smaller (132 mm for males and 145 mm for females) [25]. The Albanian population from the present study was of similar body size to the Turkish Thrace population (153 mm for males and 175 mm for females) [7]. Hermann’s tortoises of Serbia (populations of the eastern, central, and southern), however, were larger in body size than the Albanian population [17,25]. The largest Hermann’s tortoises (346 mm) were found in Bulgaria [5], whereas the smallest (153 mm in females) were found in the south of the Peloponnese in Greece [42].

These similarities or differences in body size of Hermann’s tortoises may be associated with the latitude at which each population lives because previous studies showed that Hermann’s tortoises at higher (more northern) latitudes had a larger body size. The body size of Italian Hermann’s tortoise showed variation in the north-south cline following Bergmann’s rule [22], and the body size of Hermann’s tortoises in Greece was strongly correlated with latitude [42]. Similar results were reported for *T. graeca* [27]. Additionally, some chelonians supported Bergmann’s rule, whereas some rejected it [43]. 

In our study, the CCL of females was positively correlated with latitude, but the same was not true for males. This CCL correlation may have an effect on the reproductive output of the female tortoises. The northern latitudes have a shorter reproductive season as the climate will be colder, with fewer hours of daylight per day in the colder months, thus limiting the number of successive clutches [44]. Moreover, larger females in northern latitudes lay larger clutches but produce smaller eggs [27]. In a review of the clutch size of reptiles [45], some species of tortoises had larger clutches in the north. Clutch size is also associated with female body size in Hermann’s tortoises [46]. Moreover, clutch size and egg size is negatively correlated in all tortoises [47]. As tortoises are ectotherms, it can be disadvantageous to have a larger body size because of the time required for warming up a larger mass of tissues [48]. In females of increasing body size, laying more eggs with a decreased egg size may be an effort to increase reproductive success within a shorter breeding season due to a colder climate. However, in this study, we did not investigate the reproductive output of Albanian Hermann’s tortoises and no such studies have been carried out. The effect of body size in the north-south cline on the reproductive output of Albanian tortoises should be studied in the future.

The body condition index (CI) of Hermann’s tortoises for overall Albania is zero, which means the observed body mass is equal to the predicted body mass. However, the CI showed a mean negative value in males and a mean positive value in females and was significantly different between males and females. Similar differences were also reported from France [49] and the Turkish Thrace [7]. The CI can be affected by habitat conditions, food and water availability, and tortoise activities [35,49]; it increases in spring and decreases in summer [49]. Our study was conducted during the mating season, and males tend to feed less frequently during this time. Also, the CI showed geographic variations where the central part of Albania (Tirana and Berati) had a positive value, whereas the other regions had a negative value. This difference may be related to the stability of the environmental factors of each region, such as habitat conditions and/or food and water availability. However, the geographical location within Greece did not affect the mass-to-length relationship and CI of Hermann’s tortoises. In addition, perhaps the fact that females are naturally and adaptively heavier and larger than males may have led to the difference in CI. This may also represent the result of adaptation between the sexes or regions. For instance, it was reported that a 5% difference in SCL for sex or regions might lead to a 12–15% difference in predicted M’, which could change the CI by 0.05–0.06 [35]. However, it was stated that females being heavier reflected sexual shape dimorphism, and such mass differences due to morphology should be defined in terms of relative mass, not body condition [34].

Hermann’s tortoises showed regional morphological differences in Albania. As a result of these differences, three groups (northern, central, and southern) evolved. These three groups were Shkodra in the north, Tirana, Ballshi, and Berati in the center, and Saranda in the south. The characteristics that played a role in the morphological separation of regions were generally carapace measurements (SCW, CCW, SCW6, and SCL). Similar regional differences were reported between tortoise populations in Serbia and Montenegro, and the Montenegrin population was characterized by small body size and mass [29]. The Hermann’s tortoise populations of the Trebinje (Bosnia and Herzegovina) and Pčinja river valley (Serbia) were distinguished from each other based on carapace shape analyses with geometric morphometry [26].

Regional differences in morphology may be due to the survival rate, growth rate, mortality rate, and genetics of the populations, or north-south cline (i.e., Bergmann’s rule). Variation in adult survival rates would be sufficient to explain differences in adult size among populations in Greece [50]. This means larger tortoises might have a higher survival rate and may explain why some populations show variations in size. Although the adult survival rate is negatively correlated with growth rate, variation in growth rate in the population limits variation in adult size. Differences in adult mortality rates among the populations may also explain variation in adult size. This situation may be due to the high environment temperature caused by frequent fires [42] or due to predation [51]. The low or high mortality rate affects the number of adult individuals leaving the population, and this affects the mean body size of the population. The effect of mortality rate on the mean size of the adult population was reported for other chelonian species like sea turtles [52]. In this context, we can say that Saranda and Shkodra populations, which are smaller in size and less heavy in mass, may have a low survival rate; that is, a high mortality rate. This situation may have had an effect on the average morphology, and the populations may have been separated from each other and the other regions this way. In other words, we can say that Berati, Tirana, and Ballshi populations may have similar survival rates and mortality rates. From a different point of view, the allometric growth effect may explain the separation of the Berati, Tirana, and Ballshi populations in the center, the Saranda population in the south, and the Shkodra population in the north. The presence of allometric growth can affect growth rate and mortality or survival rates, which may affect their morphology. The allometric growth has been previously reported for different populations of Hermanni tortoises [26,39,53]. However, its effect on population divergence is limited based on both carapace and plastron shape analysis [26,53]. We did not examine the allometry of Hermanni tortoises of Albania in the present study. We recommend this topic should be investigated in Albania in the future.

Another reason for regional differences may be due to molecular differences, although we did not examine this factor in the present study. Two populations of Hermann’s tortoises were distinguished genetically between Trebinje (Bosnia and Herzegovina) and the Pčinja river valley of Serbia [26]. Molecular variations may have been influenced by the biogeographical history of southern Europe and geological barriers such as the Dinaric Alps and other mountain systems that may have divided the populations. In Albania, although there are some geographical barriers between the regions of this study (such as the large rivers Mati, Shkumbini, Semani, and Vjosa), we do not think that these barriers completely separate the regions. We hypothesize that the morphological changes of tortoises may have been caused by environmental conditions of the regions, the availability of food and water, or other factors. Morphological variations may be the result of phenotypic flexibility, which is related to environmental conditions and plays a major role in the external morphology of tortoises.

## 5. Conclusions

Female Albanian Hermann’s tortoises were found to be larger and heavier than males, a finding that was compatible with previous studies. The existence of a female-biased population was determined, and the Albanian population was morphologically divided into three groups (northern, central, and southern). Among the reasons for this divergence, besides the phenotypic flexibility caused by environmental conditions, may have been the survival and mortality rates of the Hermann’s tortoise populations in each region of Albania. This study has paved the way for future investigations concerning the population status of the species, such as recruitments to the population, by determining reproductive output, clutch size, and its relationship with latitude. In particular, our findings strongly suggest that the molecular variation and reproductive output of the Albanian Hermann’s tortoise should be investigated in the near future.

## Figures and Tables

**Table 1:** Descriptive statistics of the body condition index (CI) of Hermann’s tortoises in five regions of Albania.

Regions	n	Mean ± Std Dev	Min–Max
Shkodra	21	−0.01 ± 0.13	−0.19–0.43
Tirana	40	0.03 ± 0.06	−0.06–0.36
Berati	53	0.00 ± 0.08	−0.23–0.19
Ballshi	25	−0.02 ± 0.04	−0.10–0.08
Saranda	49	−0.02 ± 0.12	−0.22–0.61
Total	188	0.00 ± 0.09	−0.23–0.61

**Table 2:** Percentage of initial classifications that were correct, shown by region.

Regions	Berati	Shkodra	Saranda	Ballshi	Tirana	Total
Berati	**69.8**	0.0	0.0	5.7	24.5	100.0
Shkodra	0.0	**100.0**	0.0	0.0	0.0	100.0
Saranda	0.0	0.0	**100.0**	0.0	0.0	100.0
Ballshi	4.0	0.0	0.0	**68.0**	28.0	100.0
Tirana	7.5	0.0	0.0	10.0	**82.5**	100.0

Bold indicates the percentage of individuals in each region correctly classified into their population

## Data Availability

Not applicable.

## Supplementary Materials

The following are available online at https://www.mdpi.com/2076-2615/11/1/134/s1, Table S1: Descriptive statistics (mean ± standard deviation) of morphological dimensions‡ of Albanian Hermann’s tortoises stratified by region and sex.
