# Supplementary material for: Morphological Divergence of Hermann’s Tortoise (Testudo hermanni boettgeri Mojsisovits, 1889) in Albania
Source: Animals (Basel). 2021 Jan 9;11(1):134. doi: 10.3390/ani11010134 (PMC7827169; doi:10.3390/ani11010134)
Supplement: Supplementary file 1 [file animals-11-00134-s001.pdf]

Supplement Table S1. Descriptive statistics (mean ± standard deviation) of morphological dimensions‡ of Albanian Hermann's tortoises stratified by region and sex.

Dimensions	Region	Male			Female			Combined Total		
		N	Mean ±Sd	Min-Max	N	Mean±Sd	Min-Max	N	Mean±Sd	Min-Max
Mass	Shkodra	14	645.7 ±111.9	450-870	7	838.6 ±552.6	250-1550	21	710±329.3	250-1550
	Tirana	14	878.6 ±274.0	242-1183	26	1306.2 ±264.7	539-1860	40	1156.6±335.6	242-1860
	Berati	13	688.8 ±276.9	300-1182	40	1090.6 ±351.4	300-1850	53	992±375.2	300-1850
	Ballshi	18	813.9 ±227.1	350-1250	7	1071.4 ±494.9	310-1920	25	886±334.2	310-1920
	Saranda	22	663.4 ±227.7	160-1150	27	1104.7 ±279.3	500-1620	49	906.6±337.8	160-1620
	Total	81	735.0 ±241.8	160-1250	107	1128.8 ±355.6	250-1920	188	959.2±367.3	160-1920
SCL	Shkodra	14	145.4 ±13.8	130-170	7	155.7 ±42.4	100210	21	148.8±26.2	100-210
	Tirana	14	154.8 ±20.8	101-174	26	182.0 ±13.1	139-207	40	172.5±20.7	101-207
	Berati	13	148.5 ±18.3	112-177	40	166.3 ±19.7	124-212	53	161.9±20.7	112-212
	Ballshi	18	159.0 ±16.3	121-185	7	169.1 ±32.1	112-216	25	161.8±21.6	112-216
	Saranda	22	148.3 ±19.1	100-190	27	177.4 ±22.1	120-220	49	164.4±25.3	100-220
	Total	81	151.3 ±18.2	100-190	107	172.4 ±22.9	100-220	188	163.3±23.4	100-220
CCL	Shkodra	14	193.2 ±15.6	170-225	7	200.0 ±44.0	140-250	21	195.5±27.4	140-250
	Tirana	14	203.8 ±27.3	131-231	26	227.8 ±15.2	177-255	40	219.4±23.1	131-255
	Berati	13	192.8 ±23.9	149-238	40	209.1 ±24.6	155-253	53	205.1±25.2	149-253
	Ballshi	18	210.1 ±21.2	160-245	7	211.1 ±36.0	142-260	25	210.4±25.3	142-260
	Saranda	22	182.7 ±21.8	135-225	27	212.1 ±32.1	130-320	49	198.9±31.4	130-320
	Total	81	195.9 ±23.9	131-245	107	214.0 ±28.0	130-320	188	206.2±27.8	130-320
SCW	Shkodra	14	120.0 ±9.0	100-130	7	124.3 ±25.1	90-150	21	121.4±15.7	90-150
	Tirana	14	133.4 ±21.3	83-155	26	141.3 ±11.6	103-165	40	138.5±15.9	83-165
	Berati	13	115.6 ±15.6	88-140	40	124.4 ±14.1	95-150	53	122.3±14.9	88-150
	Ballshi	18	129.3 ±13.8	100-153	7	131.6 ±22.9	93-168	25	129.9±16.4	93-168
	Saranda	22	167.9 ±31.6	110-206	27	167.5 ±41.0	120-250	49	167.7±36.7	110-250
	Total	81	136.7 ±28.8	83-206	107	139.9 ±29.8	90-250	188	138.5±29.3	83-250
CCW	Shkodra	14	183.9 ±15.3	155-215	7	200.0 ±53.2	130-260	21	189.3±32.6	130-260
	Tirana	14	335.1 ±41.3	230-383	26	383.0 ±27.9	282-435	40	366.2±40.0	230-435
	Berati	13	310.3 ±38.8	233-374	40	346.3 ±37.5	264-420	53	337.4±40.6	233-420

	**Ballshi**	18	329.6 ±32.6	257-380	7	340.7 ±57.7	238-428	25	332.7±40.1	238-428
	**Saranda**	22	279.7 ±64.2	170-353	27	266.3 ±76.2	160-410	49	272.3±70.7	160-410
	**Total**	81	288.7 ±68.0	155-383	107	325.1 ±73.5	130-435	188	309.4±73.2	130-435
**PL**	**Shkodra**	14	96.1 ±12.3	70-115	7	125.7 ±36.2	75-265	21	106.0±26.4	70-165
	**Tirana**	14	138.9 ±21.2	90-180	26	162.2 ±12.0	119-181	40	154.1±19.2	90-181
	**Berati**	13	119.8 ±14.9	94-144	40	147.4 ±23.2	106-245	53	140.6±24.5	94-245
	**Ballshi**	18	134.4 ±12.0	111-155	7	157.1 ±24.1	113-193	25	140.8±18.9	111-193
	**Saranda**	22	123.0 ±13.9	91-160	27	158.8 ±18.2	110-190	49	142.7±24.2	91-190
	**Total**	81	123.1 ±20.3	70-180	107	153.1 ±22.6	75-245	188	140.2±26.2	70-245
**H**	**Shkodra**	14	79.6 ±6.0	70-90	7	87.1 ±22.3	60-115	21	82.1±13.7	60-115
	**Tirana**	14	77.8 ±10.0	54-91	26	92.4 ±7.0	69-107	40	87.3±10.7	54-107
	**Berati**	13	78.2 ±9.4	59-91	40	87.7 ±8.1	65-103	53	85.4±9.3	59-103
	**Ballshi**	18	83.3 ±8.3	64-95	7	86.3 ±14.4	57-102	25	84.1±10.2	57-102
	**Saranda**	22	72.3 ±13.4	40-91	27	90.3 ±12.1	65-120	49	82.2±15.5	40-120
	**Total**	81	77.9 ±10.6	40-95	107	89.4 ±10.8	57-120	188	84.4±12.1	40-120
**G**	**Shkodra**	14	20.9 ±3.9	10-25	7	20.0 ±7.1	15-35	21	20.6±5.0	10.0-35
	**Tirana**	14	17.2 ±3.4	10-21	26	21.6 ±2.9	15-28	40	20.1±3.7	10.0-28
	**Berati**	13	16.6 ±2.8	14-23	39	20.3 ±2.7	15-31	52	19.3±3.1	14.0-31
	**Ballshi**	18	16.4 ±3.6	9-25	7	18.1 ±2.8	12-20	25	16.9±3.4	9.0-25
	**Saranda**	22	14.7 ±3.3	10-20	27	18.6 ±3.9	10-26	49	16.8±4.1	10.0-26
	**Total**	81	16.9 ±3.9	9-25	106	20.1 ±3.6	10-35	187	18.6±4.1	9.0-35
**SHL**	**Shkodra**	14	19.6 ±3.7	10-25	7	21.4 ±8.5	10-30	21	20.2±5.6	10-30
	**Tirana**	14	23.7 ±4.0	16-28	26	27.1 ±3.6	16-33	40	25.9±4.0	16-33
	**Berati**	13	23.2 ±2.9	19-28	40	23.9 ±4.2	16-38	53	23.7±3.9	16-38
	**Ballshi**	18	25.3 ±1.9	22-29	7	27.4 ±6.2	20-40	25	25.9±3.6	20-40
	**Saranda**	22	21.7 ±4.0	15-30	27	26.0 ±4.2	12-32	49	24.1±4.6	12-32
	**Total**	81	22.7 ±3.8	10-30	107	25.3 ±4.8	10-40	188	24.2±4.6	10-40
**SPL**	**Shkodra**	14	10.6 ±2.5	5-15	7	14.3 ±8.4	5-30	21	11.8±5.3	5-30
	**Tirana**	14	9.0 ±2.1	5-12	26	12.3 ±2.8	8-20	40	11.2±3	5-20
	**Berati**	13	8.9 ±1.3	6-11	40	12.9 ±3.0	9-23	53	11.9±3.2	6-23

	**Ballshi**	18	9.4 ±1.6	6-13	7	14.0 ±1.5	11-15	25	10.7±2.6	6-15
	**Saranda**	22	10.9 ±3.4	6-19	27	14.5 ±3.6	10-20	49	12.9±3.9	6-20
	**Total**	81	9.9 ±2.5	5-19	107	13.3 ±3.6	5-30	188	11.8±3.6	5-30
**SFL**	**Shkodra**	14	14.9 ±3.1	8-20	7	19.3 ±7.3	10-30	21	16.4±5.2	8-30
	**Tirana**	14	10.9 ±2.1	6-14	26	11.4 ±1.9	8-16	40	11.2±2	6-16
	**Berati**	13	9.9 ±3.6	6-19	40	10.7 ±3.0	6-23	53	10.5±3.2	6-23
	**Ballshi**	18	10.2 ±2.3	7-14	7	11.4 ±3.8	6-17	25	10.5±2.8	6-17
	**Saranda**	22	9.0 ±1.9	5-13	27	10.0 ±2.4	5-15	49	9.6±2.2	5-15
	**Total**	81	10.8 ±3.2	5-20	107	11.3 ±3.8	5-30	188	11.1±3.5	5-30
**ANSS**	**Shkodra**	14	30.0 ±5.5	20-40	7	29.3 ±10.6	15-40	21	29.8±7.3	15-40
	**Tirana**	14	28.4 ±6.3	14-35	25	30.4 ±4.3	15-35	39	29.6±5.1	14-35
	**Berati**	13	30.2 ±6.7	16-40	40	30.3 ±5.6	22-48	53	29.9±6.9	2-48
	**Ballshi**	18	30.1 ±7.3	15-48	7	27.9 ±11.2	13-43	25	41.4±61.7	13-335
	**Saranda**	22	30.0 ±7.8	13-45	27	34.4 ±6.6	22-45	49	32.4±7.4	13-45
	**Total**	81	29.7 ±6.7	13-48	106	31.1 ±6.6	13-48	187	32.0±23.4	2-335
**AN**	**Shkodra**	14	44.6 ±10.3	20-60	7	36.4 ±16.5	20-60	21	41.9±12.9	20-60
	**Tirana**	14	51.3 ±10.7	28-72	26	39.6 ±5.1	29-51	40	43.7±9.3	28-72
	**Berati**	13	44.3 ±9.8	27-58	40	35.3 ±5.1	22-47	53	37.5±7.6	22-58
	**Ballshi**	18	49.7 ±6.2	35-61	7	34.4 ±7.9	22-48	25	45.4±9.6	22-61
	**Saranda**	22	44.0 ±6.8	27-55	27	38.1 ±4.4	30-47	49	40.7±6.3	27-55
	**Total**	81	46.7 ±8.9	20-72	107	37.0 ±6.6	20-60	188	41.2±9.0	20-72
**TL**	**Shkodra**	14	31.8 ±7.5	15-50	7	24.0 ±12.5	15-50	21	29.2±9.9	15-50
	**Tirana**	14	42.4 ±10.9	20-55	26	23.0 ±3.3	15-29	40	29.8±11.6	15-55
	**Berati**	8	39.6 ±7.8	30-50	32	23.3 ±4.6	17-34	40	26.6±8.5	17-50
	**Ballshi**	18	30.6 ±7.9	18-50	7	15.7 ±3.4	10-21	25	26.4±9.7	10-50
	**Saranda**	22	35.7 ±10.3	10-55	27	29.6 ±8.6	15-60	49	32.3±9.8	10-60
	**Total**	76	35.4 ±10.0	10-55	99	24.4 ±7.3	10-60	175	29.2±10.1	10-60
**SCW6**	**Shkodra**	14	121.8 ±12.5	100-150	7	118.6 ±26.1	80-150	21	120.7±17.6	80-150
	**Tirana**	14	120.2 ±17.0	83-140	26	137.5 ±10.5	100-157	40	131.4±15.4	83-157
	**Berati**	8	114.3 ±12.5	99-133	32	116.6 ±32.1	13-179	40	116.1±29.1	13-179

	**Ballshi**	18	119.2 ±12.7	95-140	7	125.6 ±22.4	88-162	25	121.0±15.8	88-162
	**Saranda**	22	105.1 ±34.9	50-150	27	98.1 ±38.6	60-165	49	101.3±36.7	50-165
	**Total**	76	115.3 ±22.7	50-150	99	117.8 ±32.2	13-179	175	116.7±28.4	13-179
	**Shkodra**	14	178.6 ±12.3	160-200	7	188.6 ±41.8	130-240	21	181.9±25.4	130-240
	**Tirana**	14	195.2 ±24.8	134-220	26	226.6 ±17.1	165-260	40	215.6±24.9	134-260
**CCW6**	**Berati**	8	201.3 ±32.7	171-271	32	217.3 ±37.7	160-379	40	214.1±36.9	160-379
	**Ballshi**	18	194.1 ±18.5	151-230	7	208.3 ±37.2	140-263	25	198.1±25.1	140-263
	**Saranda**	22	168.5 ±22.7	100-195	27	205.5 ±40.6	90-340	49	188.9±38.3	90-340
	**Total**	76	184.8 ±24.7	100-271	99	213.9 ±35.6	90-379	175	201.2±34.4	90-379
	**Shkodra**	14	115.4 ±8.9	100-130	7	137.1 ±33.9	95-180	21	122.6±22.5	95-180
	**Tirana**	14	118.0 ±12.9	84-134	26	151.2 ±12.4	112-172	40	139.6±20.3	84-172
**MPL**	**Berati**	8	114.1 ±12.2	96-130	32	139.0 ±13.7	110-173	40	134.1±16.7	96-173
	**Ballshi**	18	121.3 ±10.6	102-142	7	148.4 ±23.8	103-182	25	128.9±19.4	102-182
	**Saranda**	22	109.3 ±13.1	80-150	27	144.6 ±21.1	79-170	49	128.8±25.1	79-170
	**Total**	76	115.4 ±12.3	80-150	99	144.3 ±18.7	79-182	175	131.7±21.6	79-182
	**Shkodra**	14	25.2 ±3.7	20-30	7	29.3 ±9.3	20-40	21	26.6±6.2	20-40
	**Tirana**	14	30.7 ±3.1	23-35	26	36.4 ±3.0	26-42	40	34.4±4.0	23-42
**WV2**	**Berati**	8	32.5 ±2.2	30-36	32	36.8 ±4.8	29-51	40	36.0±4.7	29-51
	**Ballshi**	17	30.6 ±3.0	26-36	7	36.6 ±5.3	27-42	24	32.4±4.6	26-42
	**Saranda**	21	31.4 ±6.4	20-42	27	35.8 ±5.0	21-42	48	33.9±6.0	20-42
	**Total**	74	30.0 ±4.9	20-42	99	35.9 ±5.2	20-51	173	33.4±5.8	20-51
	**Shkodra**	14	30.4 ±4.6	25-40	7	33.6 ±10.3	20-45	21	31.4±6.9	20-45
	**Tirana**	14	35.3 ±4.1	24-40	26	42.2 ±3.7	30-50	40	39.8±5.1	24-50
**WV3**	**Berati**	8	37.3 ±5.3	33-49	32	43.0 ±9.7	32-90	40	41.9±9.2	32-90
	**Ballshi**	17	35.1 ±3.5	30-41	7	40.4 ±7.3	28-50	24	36.6±5.4	28-50
	**Saranda**	20	36.5 ±5.1	26-48	27	41.4 ±5.2	26-50	47	39.3±5.7	26-50
	**Total**	73	34.8 ±5.0	24-49	99	41.5 ±7.4	20-90	172	38.7±7.3	20-90
	**Shkodra**	14	28.6 ±4.3	20-36	7	28.6 ±7.5	20-40	21	28.6±5.3	20-40
**WV4**	**Tirana**	14	36.1 ±5.4	20-41	25	39.2 ±4.1	30-48	39	38.1±4.8	20-48
	**Berati**	8	35.3 ±5.1	29-42	32	36.6 ±4.4	27-49	40	36.4±4.5	27-49

**Ballshi**	17	34.6 ±4.6	26-42	7	35.3 ±7.3	23-46	24	34.8±5.4	23-46
**Saranda**	20	32.7 ±6.5	19-46	27	36.4 ±5.5	22-46	47	34.8±6.2	19-46
**Total**	73	33.29 ±5.7	19-46	98	36.6 ±5.6	20-49	171	35.2±5.9	19-49

‡ SCL denotes straight carapace length; CCL denotes curved carapace length; SCW denotes straight carapace width, CCW denotes curved carapace width or maximal perimeter side to side; PL denotes plastron length; H denotes maximal height; G denotes inter gular suture length; SHL denotes inter humeral suture length; SPL denotes inter pectoral suture length; SFL denotes inter femoral suture length; ANSS denotes distance from the anal notch to the supracaudal scute tip; AN denotes the anal notch between the two anal scutes; TL denotes tail length from the cranial margin of the cloaca to the tail´s tip; SCW6 denotes the carapace width at the level of the 6th marginal scute; CCW6 denotes the curved carapace width at the level of the 6th marginal scute; PML denotes the plastron midline length from the gular notch to the anal notch; WV2 denotes the maximal width of 2nd vertebral; WV3 denotes the maximal width of the 3rd vertebral; WV4 denotes the maximal width of 4th vertebral
